# Copy number variation in the *CES1* gene and the risk of non-alcoholic fatty liver in a Chinese Han population

**DOI:** 10.1038/s41598-021-93549-2

**Published:** 2021-07-07

**Authors:** Bing bing Chen, Jian hui Yan, Jing Zheng, He wei Peng, Xiao ling Cai, Xin ting Pan, Hui quan Li, Qi zhu Hong, Xian-E Peng

**Affiliations:** 1 Department of Epidemiology and Health Statistics, Fujian Provincial Key Laboratory of Environment Factors and Cancer, School of Public Health, Fujian Medical University, Fujian, 350122 China; 2 Department of Hospital Infection Control, First Hospital of Quanzhou Affiliated to Fujian Medical University, Quanzhou, China; 3 Department of Infectious Disease, The Second Affiliated Hospital of Hainan Medical College, Haikou, China; 4 Key Laboratory of Ministry of Education for Gastrointestinal Cancer, Fujian Medical University, Fujian, China

**Keywords:** Genetics, Diseases

## Abstract

A recent genome-wide copy number variations (CNVs) scan identified a 16q12.2 deletion that included the carboxylesterase 1 (*CES1*) gene, which is important in the metabolism of fatty acids and cholesterol. We aimed to investigate whether CES1 CNVs was associated with susceptibility to non-alcoholic fatty liver disease (NAFLD) in a Chinese Han population. A case–control study was conducted among 303 patients diagnosed with NAFLD and 303 age (± 5) and sex-matched controls from the Affiliated Nanping First Hospital of Fujian Medical University in China**.** The copy numbers of *CES1* were measured using TaqMan quantitative real-time polymerase chain reaction (qPCR) and serum CES1 was measured using enzyme-linked immunosorbent assays. The Chi-squared test and a logistic regression model were used to evaluate the association between *CES1* CNVs and NAFLD susceptibility. The distribution of *CES1* CNVs showed a higher frequency of CNVs loss (< 2) among patients; however, the difference was not significant (*P* = 0.05). After controlling for other known or suspected risk factors for NAFLD, *CES1* CNVs loss was significantly associated with greater risk of NAFLD (adjusted OR = 2.75, 95% CI 1.30–5.85, *P* = 0.01); while CES1 CNVs gain (> 2) was not. There was a suggestion of an association between increased CES1 serum protein levels and CNVs losses among cases, although this was not statistically significant (*P* = 0.07). Copy number losses (< 2) of *CES1* contribute to susceptibility to NAFLD in the Chinese Han population.

## Introduction

The worldwide prevalence of non-alcoholic fatty liver disease (NAFLD) is about 25%, ranging from 13% in Africa to 32% in the Middle East, and is now a leading cause of chronic liver disease^1^. Although the disease is relatively benign in the early stages, when severe clinical forms, such as non-alcoholic steatohepatitis, cirrhosis, and even hepatocellular carcinoma (HCC) occur, the long-term prognosis worsens^2^. However, the specific processes responsible for the development and progression of NAFLD remain largely unknown. Hence, determining the pathogenesis and predisposing factors of NAFLD is crucial to understand its biology. A growing body of evidence indicates that NAFLD develops as a result of a complex process involving many factors, including genetic susceptibility and environmental insults^3^.

The role of genetic variations in NAFLD, specifically single nucleotide polymorphisms (SNPs), has been the focus of extensive research during the last decade. Several studies have identified multiple SNPs associated with susceptibility to NAFLD, such as those in the *PNPLA3* (patatin like phospholipase domain containing 3)^4^, *MTHFR* (methylenetetrahydrofolate reductase)^5^, *MTTP* (microsomal triglyceride transfer protein)^6^, and *APPL1* (adaptor protein, phosphotyrosine interacting with PH domain and leucine zipper 1) genes^7^. However, a previous study demonstrated that even for high familial risk diseases, such as thyroid cancer, the few significant SNPs had limited prediction power^8^. Thus, other genetic or epigenetic variations should be investigated, such as copy number variation (CNVs).

In contrast to SNPs, CNVs are a form of quantitative structural rearrangement that include deletions, duplications, and higher order amplifications at regions larger than 50 base pairs^9^. In the last decade, an increasing number of both rare and common CNVs changes have been definitively linked to a wide range of phenotypes and diseases^10^. Recently, a genome-wide association study (GWAS) identified that four rare, novel or both CNVs may influence the development of NAFLD. A deletion CNVs was noted at the 16q12.2 locus, which includes the carboxylesterase 1 (*CES1*) gene. CES1 is very important in the metabolism of fatty acids and cholesterol^11^. The expression of *CES1* is higher in human NAFLD hepatic tissue than in normal controls’ hepatic tissue^12^. Hepatic CES1 is believed to play a critical role in regulating both hepatic lipid and carbohydrate metabolism, and the concomitant control of hepatic lipid biosynthesis, secretion, and deposition^13,14^. In addition, CES1 expression correlates positively with obesity and associated cardiovascular disease risk factors^15,16^, which are also risk factors for NAFLD. Interestingly, the 16q12.2 locus harbors genetic variants associated with body mass index (BMI)^17^.

Thus, we speculated that CNVs of *CES1* might be associated with susceptibility to NAFLD. In the present study, we investigated whether *CES1* CNVs was associated with susceptibility to NAFLD in a Chinese Han population.

## Results

### Demographic and clinical data of the subjects

The subjects’ demographic and clinical data are shown in Table 1. There were 303 patients with NAFLD and 303 controls. There were statistical significant differences between the two groups with respect to age, history of hypertension and hyperlipidaemia, and ALT, AST, GGT, TC, TG, and HDL-C levels, but not with sex, educational level, occupational status, income, marital status, smoking status, tea drinking status, exercise, history of diabetes, and serum CES1 levels.

**Table 1:** General characteristics and clinical data of cases and controls, *n* (%) or medians (IQRs).

Variables	Cases (*n* = 303)	Controls (*n* = 303)	*χ*^2^ or *Z*	*P*-value*
**Sex**			0.000	1.00
Males	213 (70.30)	213 (70.30)		
Females	90 (29.70)	90 (29.70)		
**Age**			7.611	0.02
< 40	90 (29.70)	80 (26.40)		
40–60	196 (64.69)	187 (61.72)		
≥ 60	17 (5.61)	36 (11.88)		
**Educational level**			3.644	0.06
Junior middle school and less than	62 (20.46)	82 (27.06)		
Senior high school and above	241 (79.54)	221 (72.94)		
**Occupational status**			0.334	0.85
Mental labour	69 (22.77)	69 (22.77)		
Physical labor	81 (26.73)	87 (28.71)		
Other	153 (50.50)	147 (48.52)		
**Income (yuan/month)**			0.489	0.78
< 2000	1 (5.61)	16 (5.28)		
2000–3000	93 (30.69)	101 (33.33)		
≥ 3000	193 (63.70)	186 (61.39)		
**Marital status**			0.868	0.35
Single	28 (9.24)	35 (11.55)		
Married or other	275 (90.76)	268 (88.45)		
**Smoking status**			0.638	0.42
Never smoker	208 (68.65)	217 (71.62)		
Smoker	95 (31.35)	86 (28.38)		
**Teaing status**			2.009	0.16
Yes	194 (64.03)	177 (58.42)		
No	109 (35.97)	126 (41.58)		
**Exercise (times/week)**			1.961	0.16
< 3	118 (38.94)	135 (44.55)		
≥ 3	185 (61.06)	168 (55.45)		
**History of diabetes**			1.568	0.21
Yes	26 (8.58)	18 (5.94)		
No	277 (91.42)	285 (94.06)		
**History of hypertension**			17.128	< 0.001
Yes	103 (33.99)	58 (19.14)		
No	200 (66.01)	245 (80.86)		
**History of hyperlipidemia**			16.574	< 0.001
Yes	132 (43.56)	84 (27.72)		
No	171 (56.44)	219 (72.28)		
ALT (IU/L)	27.00 (21.00,40.00)	19.00 (14.00,26.00)	− 0.968	< 0.001
AST (IU/L)	23.00 (19.00,29.00)	20.00 (18.00,25.00)	− 5.014	< 0.001
GGT (IU/L)	30.00 (22.00,46.00)	22.00 (16.00,32.00)	− 7.662	< 0.001
TC (mmol/L)	5.13 (4.57,5.78)	4.91 (4.45,5.43)	− 2.643	0.01
TG (mmol/L)	1.76 (1.26,2.59)	1.12 (0.86,1.54)	− 10.416	< 0.001
HDL-C (mmol/L)	1.19 (1.03,1.37)	1.35 (1.20,1.48)	− 7.235	< 0.001
LDL-C (mmol/L)	3.12 (2.56,3.77)	3.01 (2.60,3.51)	− 1.201	0.23
CES1 (ng/ml)	13.25 (10.70,19.14)	13.13 (10.63,20.88)	− 0.235	0.81

ALT, alanine aminotransferase; AST, aspartate aminotransferase; GGT, gamma-glutamyltransferase; TC, total cholesterol; TG, triglycerides; HDL-C, high-density lipoprotein cholesterol; LDL-C, low-density lipoprotein cholesterol; CES1, carboxylesterase 1.

*P-values were calculated by using the Chi-square test for categorical variables and Mann–Whitney U test for continues variables.

### Frequency distribution of *CES1* gene copy numbers

**Figure 1:**
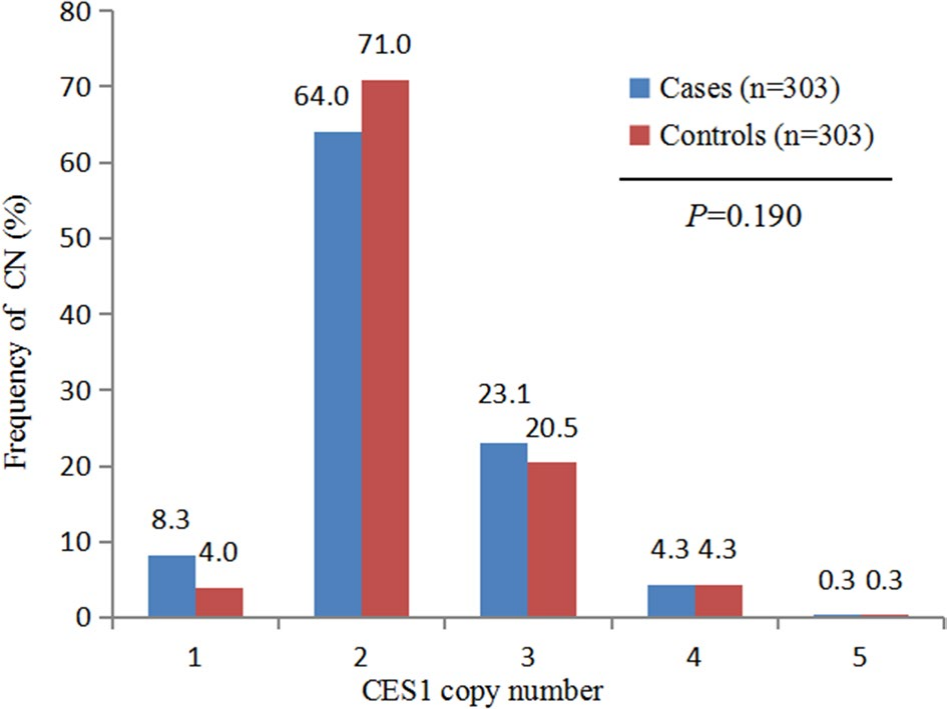
Comparison of the distribution of carboxylesterase 1 (*CES1*) copy number (CN) between subjects with and without non-alcoholic fatty liver disease (NAFLD). *P*-values above the histogram represent the Chi-squared test results.

**Figure 2:**
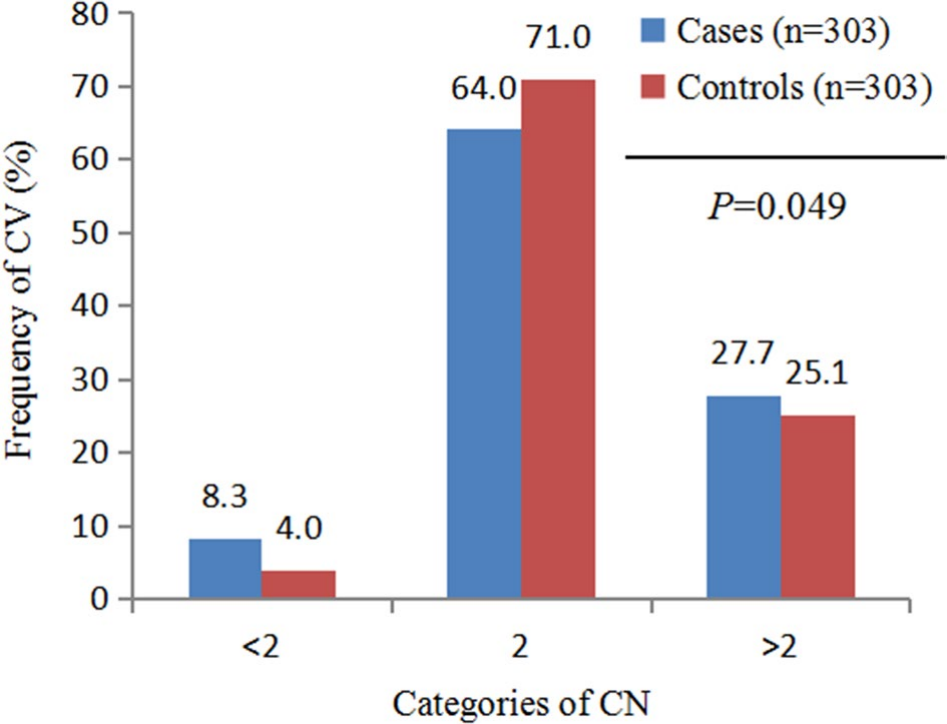
Comparison of carboxylesterase 1 (*CES1*) copy number (CN) bins (< 2, 2 and > 2) between patients with non-alcoholic fatty liver disease (NAFLD) and controls. The CN was divided into three categories by 2, *P*-values above the histogram represent the Chi-squared test results.

Figures 1 and 2 show the frequency distributions of *CES1* gene copy numbers. Overall, the copy number of the *CES1* gene ranged from two to five per diploid genome. The median number of CES1 genes was two copies. We divided the samples into copy loss (< 2), copy neutral (= 2), and copy gain (> 2) groups. There was no statistically significant difference between patients and controls in terms of copy number when assessed using the Chi-square test (*P* = 0.190, Fig. 1). After classification of copy numbers, there was a suggestion of a higher frequency of CNVs loss among patients than among controls (*P* = 0.049, Fig. 2).

### Association of *CES1* CNVs with NAFLD

As shown in Table 2, after adjusting for other potential predictors and confounders, the results of logistic regression revealed that compared with copy neutral (= 2), *CES1* copy number loss (< 2) was significantly associated with increased risk of NAFLD (adjusted OR = 2.75, 95% CI 1.30–5.85, *P* = 0.01); however, copy number gain (> 2) was not significantly associated with NAFLD disease susceptibility (adjusted OR = 1.27, 95% CI 0.86–1.86, *P* = 0.23).

**Table 2:** Association of CES1 copy numbers with NAFLD.

Variable	Cases ( *n* = 303)	Controls (*n* = 303)	Unadjused		Adjused*	
			*P*-value	OR(95% CI)	*P*-value	OR(95% CI)
**CES1 copy number**						
Neutral	194	215	–	1	–	1
Losses	25	12	0.02	2.31 (1.13,4.72)	0.01	2.75 (1.30,5.85)
Gains	84	76	0.28	1.23 (0.85,1.77)	0.23	1.27 (0.86,1.86)

CES1, carboxylesterase 1; NAFLD; non-alcoholic fatty liver disease.

*Adjusted for age, sex, educational level, occupational status, income, marital status, smoking status, tea drinking status, exercise, history of diabetes, hyperlipidaemia, and hypertension.

### Stratified analyses

**Figure 3:**
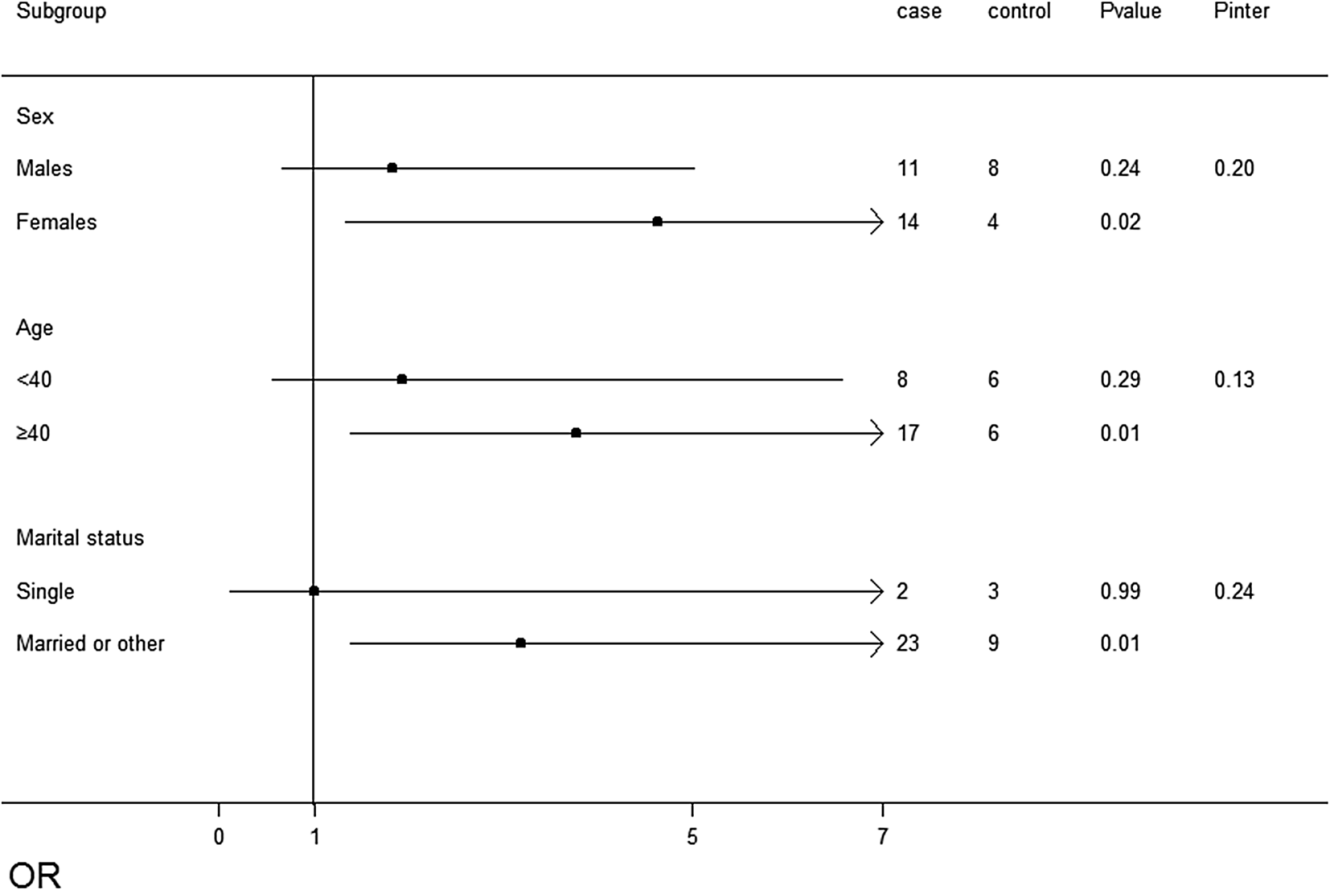
Association tests of copy number variation (CNVs) losses in patients with non-alcoholic fatty liver disease (NAFLD) across strata for various factors. The Forest plot represents the odds ratios (ORs) of the comparison of carboxylesterase 1 (*CES1*) CNVs losses versus the neutral CNVs, adjusting for age, sex, education level, occupational status, income, marital status, smoking status, tea drinking status, exercise, history of diabetes, hyperlipidaemia, and hypertension. *P*inter indicates the *P* value for the interaction between the strata and CNVs loss.

**Figure 4:**
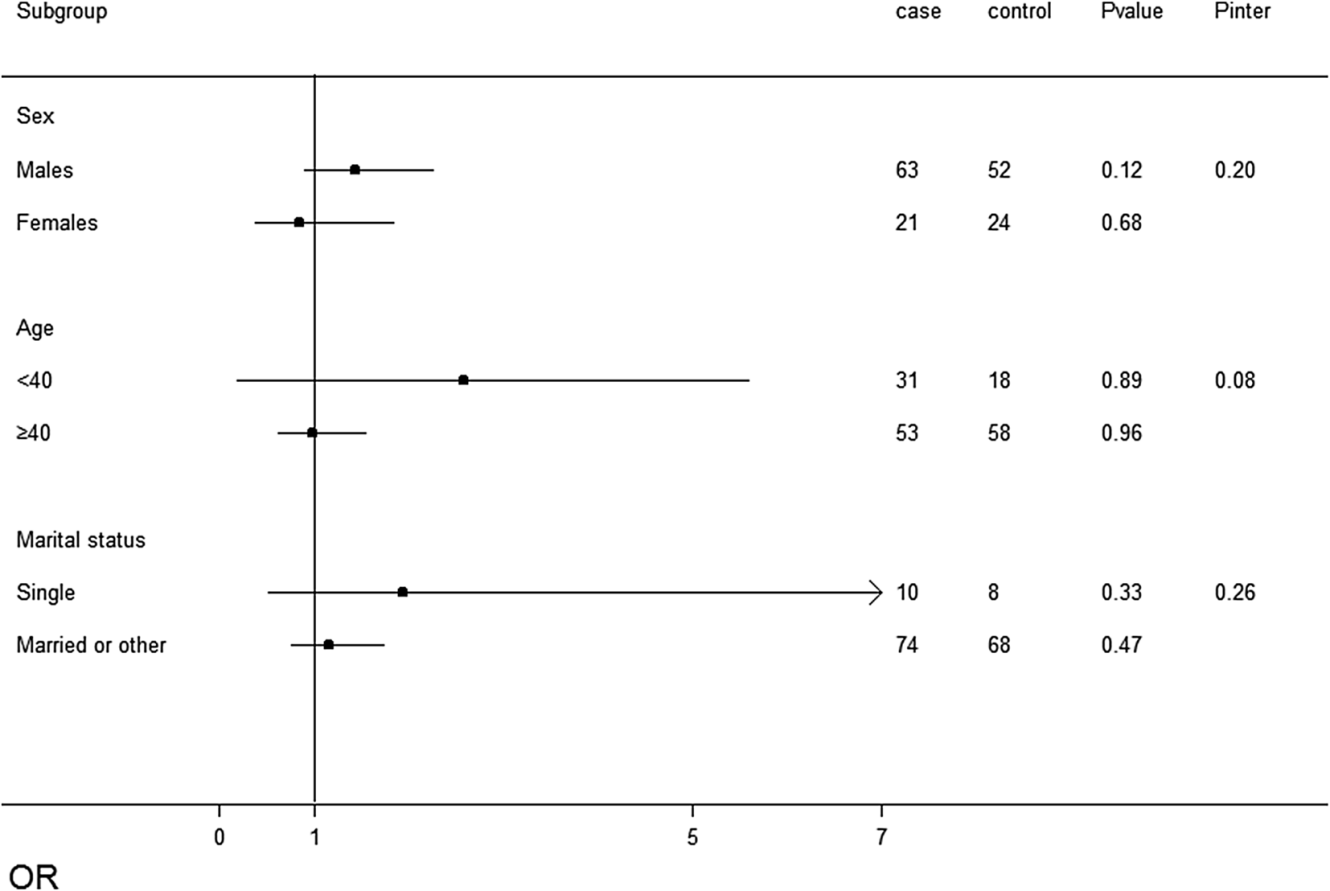
Association tests of copy number variation (CNVs) gains in patients with non-alcoholic fatty liver disease (NAFLD) across strata for various factors. The Forest plot represents the odds ratios (ORs) of the comparison of carboxylesterase 1 (*CES1*) CNVs gains versus the neutral CNVs, adjusting for age, sex, education level, occupational status, income, marital status, smoking status, tea drinking status, exercise, history of diabetes, hyperlipidaemia, and hypertension. *P*inter indicates the *P* value for the interaction between the strata and CNVs gains.

When the subjects were stratified by age, sex, and marital status, the positive association between CNVs loss and NAFLD susceptibility was consistent. CNVs loss was associated with the risk of NAFLD in women (*P* = 0.02), age more than 40 years old (*P* = 0.01), married subjects (*P* = 0.01), but not in males, age less than 40 years old, and single subjects. In the stratified levels, the association between CNVs gains and NAFLD was not significant. No significant interactions between CNVs loss/gain and potentially effect-modifying NAFLD risk factors were identified (Figs. 3, 4).

### The relationship between various clinical parameters and *CES1* CNVs

To determine whether *CES1* copy numbers were associated with specific clinical parameters of NAFLD, we compared the difference in cases between the CNVs and the AST, ALT, GGT, TC, TG, HDL-C, and LDL-C values; however, we did not find any significant differences between the clinical parameters and CES1 CNVs (Table 3).

**Table 3:** Comparison of clinical data between the CNV status among the subjects, *n* (%) or medians (IQRs).

Variables	Case (*n* = 303)				Control (*n* = 303)			*P*-value*
	Losses (*n* = 25)	Neutral (*n* = 194)	Gains (*n* = 84)	*P*-value*	Losses (*n* = 12)	Neutral (*n* = 215)	Gains (*n* = 76)	
ALT (IU/L)	28.00 (17.50, 42.50)	27.00 (21.00, 38.25)	31.00 (23.00, 42.75)	0.17	20.50 (16.25, 33.75)	19.00 (14.00, 26.00)	18.50 (15.00, 26.75)	0.38
AST (IU/L)	23.00 (18.00, 31.50)	22.00 (19.00, 28.00)	24.00 (20.00, 31.00)	0.12	24.50 (20.00, 30.75)	20.00 (17.00, 24.00)	21.00 (18.00, 27.00)	0.06
GGT (IU/L)	27.00 (21.00, 37.00)	30.00 (22.00, 46.00)	34.00 (24.00, 47.75)	0.27	21.50 (13.25, 28.75)	22.00 (16.00, 32.00)	24 (17.00, 33.50)	0.70
TC (mmol/L)	4.96 (4.30, 5.63)	5.09 (4.50, 5.88)	5.17 (4.60, 5.73)	0.72	4.68 (4.21, 5.12)	4.94 (4.49, 5.43)	4.92 (4.37, 5.44)	0.37
TG (mmol/L)	1.48 (1.29, 2.10)	1.82 (1.29, 2.69)	1.76 (1.20, 2.48)	0.35	1.15 (0.81, 1.51)	1.12 (0.88, 1.48)	1.12 (0.86, 1.44)	0.92
HDL-C (mmol/L)	1.17 (0.97, 1.30)	1.21 (1.04, 1.37)	1.19 (0.98, 1.39)	0.58	1.33 (1.11, 1.51)	1.35 (1.20, 1.48)	1.37 (1.20, 1.48)	0.74
LDL-C (mmol/L)	3.09 (2.52, 3.75)	3.12 (2.54, 3.80)	3.14 (2.63, 3.65)	0.83	2.75 (2.40, 3.32)	3.03 (2.61, 3.49)	2.99 (2.65, 3.57)	0.40
CES1 (ng/ml)	17.95 (11.91, 27.97)	13.25 (10.61, 18.65)	13.25 (10.42, 17.68)	0.07	12.88 (8.28, 17.03)	13.13 (10.50, 21.41)	13.13 (10.73, 20.99)	0.54

CNVs, copy number variation; IQR, interquartile range; ALT, alanine aminotransferase; AST, aspartate aminotransferase; GGT, gamma-glutamyltransferase; TC, total cholesterol; TG, triglycerides; HDL-C, high-density lipoprotein cholesterol; LDL-C, low-density lipoprotein cholesterol; CES1, carboxylesterase 1.

*P-values were calculated by using the Chi-square test for categorical variables and Kruskal Wallis test for continues variables.

### The effect of CES1 CNVs on serum CES1 levels

There was a suggestion of an association between increased CES1 serum protein levels and CNVs losses, although this was not statistically significant (CNVs loss, 17.95 ng/mL; CNVs neutral, 13.25 ng/mL; and CNVs gain, 13.25 ng/mL) (Table 3).

## Discussion

The results of the present study provided evidence that the loss of copy numbers of *CES1* was significantly associated with increased susceptibility to NAFLD in a Chinese Han population. Moreover, this association seemed to be independent of other predictors and lifestyle factors. The effect of *CES1* CNVs was maintained across other known or suspected risk factors for NAFLD. Furthermore, no significant interactions between CNVs and the potential modifying effects of NAFLD risk factors were identified. To the best of our knowledge, this is the first study to demonstrate a relationship between *CES1* CNVs and NAFLD susceptibility.

Common CNVs represent an important source of genetic diversity; however, their influence on phenotypic variability and disease susceptibility remains poorly understood. Recently, CNVs was recognized as a common form of human genetic variation and a contributor to a range of common diseases, such as type 2 diabetes^18^, obesity^19^, and hepatocellular carcinoma (HCC)^20^. A recent genome-wide CNVs scan identified a deletion CNVs at the 16q12.2 locus that was associated with NAFLD^21^. This region includes the *CES1* gene, which plays an important role in regulating lipid synthesis, secretion, and energy metabolism^22,23^. Although most CNVs are gene dosage insensitive^24^, in about 10% of cases, a negative correlation between CNVs and levels of gene expression is reported, i.e., these human genomes are dosage reversed^25,26^. This may be caused by reduced transcription and gene silencing as a consequence of gene duplication^24^. In the present study, we demonstrated that *CES1* CNVs losses were associated with increased risk of NAFLD. Interestingly, our data suggested that an increase in serum CES1 levels were associated with *CES1* CNVs losses, although this was not statistically significant.

A recent study showed that the expression of *CES1* correlated positively with increased lipid storage and the plasma lipid concentration, and attenuation of human *CES1* activity was beneficial for hepatic lipid metabolism. The mechanism was determined to include decreased very-low density lipoprotein secretion, decreased expression of hepatic lipogenic genes, and increased fatty acid oxidation^27^. Another study demonstrated that inactivation of mouse *Ces1d *in vivo prevented high-fat diet-induced steatosis and steatohepatitis development because of decreased de novo lipogenesis, increased fatty acid oxidation, and attenuated inflammation^28^. The attenuated steatohepatitis observed in the *Ces1d* deficient mice was accompanied by decreased inflammation and oxidative stress^28^. Furthermore, global *Ces1d* deficient mice presented improved glucose tolerance and insulin sensitivity in both chow- and high-fat/high-cholesterol diet-fed conditions^29^. However, hepatic expression of *CES1* has beneficial effects on lipid and carbohydrate metabolism, whereas loss of hepatic *CES1* causes fatty liver^14^. Similarly, another study showed that mice deficient for *CES1* became obese, even when fed on a standard chow diet, and developed hepatic steatosis and hyperlipidaemia^13^. Thus, the role of CES1 in hepatic steatosis is largely unknown.

Few studies have investigated CES1 and NAFLD in humans, thus we lack a systematic perspective about the relationship between *CES1* expression and NAFLD in humans. One study revealed elevated *CES1* expression in patients with steatosis and nonalcoholic steatohepatitis^12^. In the present study, we demonstrated that *CES1* CNVs losses might increase CES1protein levels. In addition, several studies have demonstrated that *CES1* expression correlates positively with obesity and associated cardiovascular disease risk factors^16,30,31^. One study demonstrated that the mRNA expression of *CES1* correlated positively correlated with many clinical parameters of adiposity and hypercholesterolaemia^16^. Similar upregulation of *CES1* expression in patients with obesity was reported^31^. Furthermore, positive correlations were found between different markers of metabolic syndrome (MetS) and *CES1* mRNA expression^15^. NAFLD has been regarded as the “hepatic manifestation of the metabolic syndrome”^32^. Therefore, our findings were indirectly supported by several studies that revealed a positive correlation between *CES1* expression and the risk of obesity and MetS, which share common metabolic parameters with NAFLD. This potential functional effect requires further study, particularly because we noted a tendency to increase the CES1 protein levels as the gene copy number decreased. CES1, as a novel regulator of lipogenesis in the liver, might play an important role in development NAFLD. Further studies with larger sample sizes are needed to validate the effects of *CES1* CNVs on NAFLD in various populations.

Our study has several limitations. Firstly, it is important to note that the findings observed in this study were limited to Han Chinese patients with NAFLD. Further confirmatory studies, especially populations of other ethnicities, are strongly recommended. Secondly, the relatively small number of stratified analysed samples may have resulted in the negative association in this subgroup. Thirdly, the biological functions of *CES1* CNVs in NAFLD have not been studied; thus, it is unknown how they induce or accelerate the response. Finally, the limited sample size means that our findings should be verified with larger samples in future studies.

However, this study had strengths. Firstly, the association findings were consistent with the results of a previous GWAS^11^. Secondly, extensive information on anthropometrics and lifestyle factors were collected in this study, which allowed us to adjust for confounding factors. Thirdly, the study had sufficient power to investigate interactions between CNVs and other risk factors, for which a biologically plausible mechanism might exist. Lastly, to the best of our knowledge, this is the first study to confirm the previous genome-wide report on the association of *CES1* CNVs with NAFLD.

NAFLD overemphasizes alcohol and under-emphasizes the importance of metabolic risk factors in this disease. Recently, a consensus by 32 experts from 22 countries recommended “metabolic (dysfunction) associated fatty liver disease” (MAFLD) as a more appropriate name to describe fatty liver disease associated with metabolic dysfunction, ultimately suggesting that the old acronym NAFLD should be abandoned^33,34^. Remarkably, a set of “positive” criteria to diagnose the disease—independent of alcohol intake (or misuse)—has been proposed. We believe that the proposed diagnostic criteria are novel and practical, which will help unify the terminology (e.g. for ICD-coding), to enhance the legitimacy of clinical practice and clinical trials, to improve clinical care and to move the clinical and scientific field of Hepatology forward. However, the road to implementing this change is long and will not come without major challenges. Further research will be required to evaluate the positive and negative impacts of renaming and to compare differences in clinical cohorts in whom the NAFLD versus MAFLD diagnostic criteria are applied.

## Conclusion

In conclusion, we demonstrated and confirmed the relationship between copy number variations of *CES1* and susceptibility to NAFLD. We revealed that copy number losses (< 2) of *CES1* might be a risk factor for NAFLD, and there was a suggestion that an increase in serum CES1 levels was associated with *CES1* CNVs losses, although this was not statistically significant. Determining the biological actions of CES1 will enhance our understanding of its role in NAFLD progression. Further replication and functional studies related to this finding are necessary. This study should also be replicated in a larger cohort and in populations with other ethnicities, and the association between *CES1* CNVs and MAFLD are need to be explored.

## Materials and methods

### Ethics approval and consent to participate

The study was approved by the local ethics committees of Fujian Medical University (ethics number 2014096). All methods were performed in accordance with the relevant guidelines and regulations. In addition, each subject gave written informed consent before participation in the study.

### Study design

We conducted a case–control study in a health examination centre at the Affiliated Nanping First Hospital of Fujian Medical University from April 2015 to August 2017. Patients newly diagnosed with NAFLD using ultrasonography in accordance with the “Guidelines for the diagnosis and treatment of nonalcoholic fatty liver disease in China”^35^ were included in the study. Hepatic ultrasonography examination was performed by trained ultrasonographists who were blinded to the clinical and laboratory data. Hepatic steatosis was diagnosed by characteristic echo patterns according to conventional criteria, such as the evidence of diffuse hyper-echogenicity of the liver relative to the kidneys, ultrasound beam attenuation, and poor visualization of intrahepatic structures.

### Outcome–eligibility of NAFLD cases and controls

All participants were of Chinese Han ethnicity. The exclusion criteria were as follows: (a) daily alcohol intake of > 40 g (men) and > 20 g (women); (b) a history of other liver diseases, including drug-induced liver disease, viral hepatitis, autoimmune hepatitis, total parenteral nutrition, and hepatolenticular degeneration; (c) taking hypolipidaemic or weight reduction drugs, (d) age < 18 or > 70 years old, (e) non-resident of Nanping, or (f) not of Han ethnicity.

The controls were randomly selected from the same centre during the study period. Their eligibility criteria were identical to those of the cases, except for the requirement of a diagnosis of liver steatosis; they were frequency-matched with cases by age (within 5-year intervals), gender, ethnicity, and region of origin.

### Data measurements and data collection

#### Potential confounders

Trained interviewers performed a comprehensive medical history on each participant that included eliciting information about their demographic and socio-economic characteristics (e.g., age, gender, education, income, marriage status and history of diabetes, hypertension, hyperlipidemia), lifestyle habits (e.g., smoking, drinking (alcohol), tea drinking, and physical activity), anthropometric assessment (e.g., height, body weight and blood pressure). The data were obtained from participants using structured questionnaires during face-to-face interviews.

#### Clinical data

The biochemical tests for serum concentrations of alanine aminotransferase (ALT), aspartate aminotransferase (AST), gamma-glutamyltransferase (GGT), total cholesterol (TC), triglycerides (TG), low-density lipoprotein cholesterol (LDL-C), and high-density lipoprotein cholesterol (HDL-C) of each participant were performed according to standard clinical laboratory methods carried out in an accredited laboratory at Nanping First Hospital.

### Exposure-*CES1* copy number and serum CES1 levels

#### DNA extraction

Peripheral blood was collected from all subjects in vacuum blood tubes containing EDTA-K2. Genomic DNA was isolated from 2 mL of whole blood using a whole blood genomic DNA extraction kit, according to the manufacturer’s instructions (Catalogue No DP1101/DP1102, Bioteke Corporation, Beijing, China). The concentration and purity of DNA were detected using a Beckman DU800 UV–vis spectrophotometer (Beckman, Franklin Lakes, NJ, USA) and genomic DNA was stored at − 80 °C before CNVs genotype detection.

### Taqman gene copy number assay (qPCR)

The good quality extracted DNA (OD_260_/OD_280_ = 1.7–2.0) was diluted to a final concentration of 5 ng/μL. The Applied Biosystems protocols for the TaqMan real-time quantitative polymerase chain reaction (qPCR) method were used to assess CNVs (16q12.2: Assay Hs00114970_cn) in every sample. Each reaction (20 μL) contained 10 μL of master mix, 1 μL of TaqMan Copy Number Assay, 1 μL of TaqMan Copy Number Reference Assay, 4 μL of nuclease free water, and 4 μL of genomic DNA (5 ng/μL). Quantitative real-time PCR (qPCR) was performed used the following programme: 1 PCR cycle at 95 °C for 10 min; followed by 40 cycles at 95 °C for 15 s and 60 °C for 1 min. Negative controls were introduced for every run to ensure the genotyping quality. Each sample was run in quadruplicate, and all the above reagents were obtained from Thermo Fisher Scientific (Thermo Fisher Scientific, Waltham, MA, USA). The CNVs assay was performed using an ABI 7500 Real-time PCR system. The Taqman copy number assay results were analysed using CopyCaller^`^ Software version 2.0 (Thermo Fisher Scientific). The copy numbers called with 50% confidence were included in the final analysis.

### Measurement of serum CES1 levels

Fasting blood samples were collected from each subject and then stored at − 80 °C. When performing the assay, samples were brought to room temperature. Serum CES1 levels were measured using an enzyme-linked immunosorbent assay (ELISA) (Catalogue No. ml057683; Shanghai Enzyme-linked Biotechnology Co., Ltd., Shanghai, China) according to the manufacturer’s recommendations. The intra-assay coefficient of variation (CV) of the kit was less than 10%, and the inter-assay CV was less than 12%.

### Statistical analyses

Categorical and continuous variables were compared between patients with NAFLD and controls using the Chi-squared test and the independent Mann–Whitney U test as appropriate. The distribution of *CES1* copy numbers between patients with NAFLD and control subjects was compared using the Chi-squared test.

According to our experimental results, two genomic copies of CES1 were considered to be most common in the healthy population, and therefore this copy number was set as the reference. Unconditional logistic regression models were used to compute the odds ratios (ORs) and their 95% confidence intervals (CIs) for the association between NAFLD risk and the various copy numbers of *CES1*, using the two genomic copy numbers as the reference category. The following known independent risk factors for NAFLD were included in all models: age, sex, education level, occupational status, income, marital status, smoking status, tea drinking status, exercise, history of diabetes, hyperlipidaemia, and hypertension. We evaluated the influence of CNVs status across strata of other potential predictors and confounders, comparing subjects with the CNVs loss or gain with CNVs neutral cases. We also evaluated interactions between CNVs loss or gain with age, sex, marital status. Data analyses were conducted using SPSS version 20.0.0.1 (IBM Corp., Armonk, NY, USA), and a *P* value < 0.05 was considered statistically significant.
