# Author Correction: Copy number variation in the *CES1* gene and the risk of non-alcoholic fatty liver in a Chinese Han population

**DOI:** 10.1038/s41598-022-14015-1

**Published:** 2022-06-02

**Authors:** Bing bing Chen, Jian hui Yan, Jing Zheng, He wei Peng, Xiao ling Cai, Xin ting Pan, Hui quan Li, Qi zhu Hong, Xian-E Peng

**Affiliations:** 1 Department of Epidemiology and Health Statistics, Fujian Provincial Key Laboratory of Environment Factors and Cancer, School of Public Health, Fujian Medical University, Fujian, 350122 China; 2 Department of Hospital Infection Control, First Hospital of Quanzhou Affiliated to Fujian Medical University, Quanzhou, China; 3 Department of Infectious Disease, The Second Affiliated Hospital of Hainan Medical College, Haikou, China; 4 Key Laboratory of Ministry of Education for Gastrointestinal Cancer, Fujian Medical University, Fujian, China

Correction to: *Scientific Reports* 10.1038/s41598-021-93549-2, published online 07 July 2021

The original version of this Article contained an error in the Funding section.

"This work was supported by the National Natural Science Foundation of China [Grant number: 81473047], Joint funds for the innovation of science and technology, Fujian province [Grant numbers: 2017Y9104 and 2016Y9035], the Natural Science Foundation of Fujian province [Grant number: 2019J01306]."

now reads:

"This work was supported by the National Natural Science Foundation of China [Grant number: 81473047], Joint funds for the innovation of science and technology, Fujian province [Grant numbers: 2017Y9104 and 2016Y9035], the Natural Science Foundation of Fujian province [Grant number: 2019J01316]."

The original Article has been corrected.

